# Exploring the relationship between social support, resilience, and subjective well-being in athletes of adapted sport

**DOI:** 10.3389/fpsyg.2023.1266654

**Published:** 2023-12-07

**Authors:** Tânia Mira, Miguel Jacinto, Aldo M. Costa, Diogo Monteiro, Susana Diz, Rui Matos, Raúl Antunes

**Affiliations:** ^1^Department of Sport Sciences, University of Beira Interior, Covilhã, Portugal; ^2^ISCE—Higher Institute of Lisbon and Vale do Tejo, Ramada, Portugal; ^3^ESECS—Polytechnic of Leiria, Leiria, Portugal; ^4^Life Quality Research Centre (CIEQV), Rio Maior, Portugal; ^5^Faculty of Sport Sciences and Physical Education, University of Coimbra, Coimbra, Portugal; ^6^Research Centre in Sports Sciences, Health Sciences and Human Development (CIDESD), Vila Real, Portugal

**Keywords:** subjective well-being, resilience, social support, affect, disability

## Abstract

**Introduction:**

Sports participation of people with disabilities provide an improvement in their skills, especially on access to social support, which could improve resilience and well-being. This study aims to analyze the association between types of social support (parents, coach, friend, and best friend), resilience and positive and negative affect, in 105 Portuguese athletes with disabilities aged between 13 and 61 years (32 ± 12.35 years).

**Methods:**

Participants answered a short sociodemographic questionnaire, the Portuguese version of the Positive and Negative Affect Schedule, and the Brief Resilience Scale, and a scale for assessing social support from parents, coach, friend, and best friend.

**Results:**

Social support provided by the best friend, coach, friends, and parents had a direct effect on resilience and positive and negative affect. Results showed a positive and significant association between resilience and positive affect (*r* = 0.28; *p* = 0.004) and a negative association between resilience and negative affect (*r* = −0.37; *p* ≤ 0.001). A strong relationship was found between resilience and affect, with no relationship being verified between the sources of social support and resilience or affect, as hypothesized.

**Discussion:**

For this group of athletes with disability, more than the social support they may have or may feel, resilience proved to be very important for the consequence of sports practice in terms of subjective well-being.

## Introduction

Sport is recognized as an essential toll in the education of children and young people, for the added value of physical and psychological condition provided (Conroy and Coatsworth, 2007; Macnamara and Collins, 2013). Likewise adapted sport promotes health, quality of life and social integration of people with disabilities (Blauwet and Willick, 2012; Frank et al., 2013).

The practice of sports by people with disabilities involves, in most cases, third parties (parents, friends, team-mates, coaches, among others). Social support for sport practice has proven to be fundamental. Social support is essential for well-being, it allows better integration into society and better goal realization (Banack et al., 2011). Social support refers to the providing of assistance, comfort and/or support to other people to help them cope with biological, psychological and social stress. This social support can come from any interpersonal relationship in an individual’s social network (family, friends, colleagues, coaches, among others). It can be provided in a practical (e.g., doing tasks, providing advice), tangible (e.g., giving money or other materials) and emotional way (American Psychiatric Association, 2022). Social support means an exchange of resources that takes place between at least two people, the provider, and the recipient, with the aim of improving the recipient’s well-being (Shumaker and Brownell, 1984).

Previously studies showed a positive association between the practice and the social support of parents, which have been widely studied in children, adolescents and young people (Dowda et al., 2007; Ornelas et al., 2007; Beets et al., 2010; Edwardson and Gorely, 2010; Loucaides and Tsangaridou, 2017), as well as the social support of friends (Stewart, 1993; Rodriguez and Cohen, 1998; Cheng et al., 2014; Loucaides and Tsangaridou, 2017). These two sources of social support have been presented as essential in the practice of physical activity, however the role of support coming from friends seems to have more impacting influence in this context (Cheng et al., 2014; Loucaides and Tsangaridou, 2017). The Cheng et al. (2014) study reported that the physical activity of adolescents is directly associated with the physical activity of their friends. Friendship is considered an important source of social support and influence for physical activity. Those who do physical activity tend to make friends with those who do similar amounts of physical activity, eventually imitating behaviors, creating a mutually dependent relationship between physical activity and friendship networks (Haye et al., 2011). Recent studies have looked at the social support provided by the best friend and its influence on adolescent’s practical physical activity and perceived benefits (Martin and Smith, 2002; Martin, 2006; Stearns et al., 2018; Kandola et al., 2020; Monteiro et al., 2021). On the other hand, in sports for people with disabilities context, social support is also considered to be a positive influence (Swanson et al., 2008; Machida et al., 2013; Crawford et al., 2015; Fiorilli et al., 2016; Haslett et al., 2017; Powell and Myers, 2017; Cardoso et al., 2018; Atkinson and Martin, 2020; Aitchison et al., 2021; Mira et al., 2022; Monton et al., 2022).

Coaches, parents, and friends are extremely important for their positive influence on various factors. The coach has proven to be an indispensable source of social support, offering support and guidance that results in strong relationships (Greendorfer, 2002; Jones et al., 2002; Sheridan et al., 2014; Gillham et al., 2015; Lu et al., 2016; Mira et al., 2022). Friendship is, also, considered an important source of social support and influence for sports practice. Children with disabilities usually have less friends and sport offers ample opportunity for promoting social connections (Martin and Smith, 2002; Martin, 2006). The pattern of support for athletes throughout their career should be adjusted as their needs change (Rees and Hardy, 2000).

Although social support is essential for athletes with disabilities, it is not the solution to all the challenges these athletes face, not only in their social and personal life but also in their sport, training and competition life. With many hours of training often repetitive and with implications in stress levels, time to recover from injuries that prevent them from performing and competitive anxiety with the agony of failure, athletes need not only physical resistance and talent but also mental resistance (Vallerand and Losier, 1999; Jones et al., 2002). Many studies have addressed the topic of resilience in athletes with disabilities (Machida et al., 2013; Cardoso and Sacomori, 2014; Martin et al., 2015; Porto et al., 2016; Powell and Myers, 2017; Sikorska and Gerc, 2018; Atkinson and Martin, 2020; Martin et al., 2022; Mira et al., 2022). Fletcher and Sarkar (2012) presented resilience as the set of mental and behavioral processes that promote personal assets and, in turn, protect the individual against the potential negative effects of stress. How a person reacts to adversity in a positive way depends on the adversity they have been subjected to and their own adaptation to it (Morgan et al., 2013).

Sports participation of people with disabilities has shown implication on resilience, especially on access to social support, opportunities, and meaningful social experiences (Machida et al., 2013). In a recent systematic review conducted by Mira et al. (2023), a few studies demonstrated a relationship between social support and resilience in athletes with disabilities (Machida et al., 2013; Powell and Myers, 2017; Mira et al., 2022). These results are in line with the conceptual model of sport resilience previously developed by Galli and Vealey (2008) which argues that sociocultural influences are crucial for the resilience in athletes. Just as the social support from family, coach, colleagues, and those around them, resilience is crucial to their responses to the adversity they face (Bicalho and Noce, 2019).

Fontes and Brandão (2013) reinforce the idea that resilience manifests itself throughout life from the interaction between risk and protection factors and because high performance sport is an environment that exposes athletes to risk and stress, athletes strengthen their positive personal characteristics and network of social and affective support in an effective way to overcome adversities and not abandon the career prematurely.

On the other hand, several studies have proven the role of physical activity and sport in increasing well-being (Smith et al., 2011; Mack et al., 2012; Caddick and Smith, 2014; Hogan et al., 2015) and specifically, subjective well-being (Ku et al., 2007; Downward and Rasciute, 2011; Moraes et al., 2012; Ku et al., 2014; Olsson et al., 2014). Subjective well-being is defined as the search in life for pleasure (Waterman, 2008), which represents what the person feels in relation to his/her own life (Kashdan et al., 2008). With a hedonic premise and a complex and multifaceted nature, it evaluates life cognitively and affectively, being subdivided into three components: positive affect, negative affect and satisfaction with life (Ryff and Keyes, 1995; Diener et al., 1999, 2003). Cognitive appraisals are characterized by life satisfaction and sense of personal fulfilment; affective appraisals presuppose the presence of positive affect (positive emotions and moods) and the lack of negative affect (negative emotions and moods) (Diener, 2000; Ryan and Deci, 2001; Diener et al., 2003; Diener and Ryan, 2009). People with disabilities have poorer well-being due to their characteristics and may experience anxiety and depressive disorders more often than people without disabilities (Puce et al., 2023a,b). Studies show that people with disabilities who practice sport have greater life satisfaction and well-being compared to people with disabilities who do not practice sport (Blauwet and Willick, 2012; Yazicioglu et al., 2012; Frank et al., 2013; Puce et al., 2023a,b). In a review study on this topic, it was possible to verify that the studies that analyzed subjective well-being in athletes with disabilities revealed high positive affect and low negative affect (Mira et al., 2023).

Social support and well-being are two important constructs in athletes with disabilities and their relationship has been evidenced in several studies (Crawford et al., 2015; Fiorilli et al., 2016; Haslett et al., 2017; Atkinson and Martin, 2020; Aitchison et al., 2021; Mira et al., 2022; Monton et al., 2022). Waldinger and Schulz (2016) argues that social connections are very important, people who are more socially connected are happier, healthier, and live longer. The quality of close relationships is very important and healthy relationships protect our body and brain. Good relationships keep us happier and healthier, or, in other words, a good life is built on good relationships (Waldinger and Schulz, 2016). The association between positive affect and social support from parents and friends has reinforced the importance that this support seems to have on the emotional states of athletes (VaezMousavia et al., 2013; Shapiro and Malone, 2016). The social support provided to athletes with disabilities is very important, as improvement of their career and well-being (Crawford et al., 2015; Fiorilli et al., 2016; Haslett et al., 2017; Atkinson and Martin, 2020; Aitchison et al., 2021; Mira et al., 2022; Monton et al., 2022).

At the same time, the literature has also shown a strong association between resilience and well-being in athletes with disabilities (Machida et al., 2013; Martin et al., 2015; Sikorska and Gerc, 2018; Atkinson and Martin, 2020; Martin et al., 2022; Mira et al., 2022). As argued by Fredrickson (1998), positive emotions operate as resources for coping with adversity. Subjective well-being and resilience associated with positive emotions may lead to the creation of lasting psychological resources and, consequently, greater emotional strengthening from the reinforcement of positive emotions (Fredrickson, 1998; Jaafar et al., 2014). Positive emotions lead to higher levels of resilience in the future and resilience also achieves its effects, in part, through the conception of positive emotion (Jaafar et al., 2014). Well-being sometimes results from active combat with adversity. Experiences with obstacles, failures and disappointments are necessary to know one’s own limitations and vulnerability, find internal strengths and renew resources (Fredrickson, 1998). In each risk situation, a person may react vulnerably, with a negative affect response, or resiliently, with a positive affect response.

In summary, social support is noted as one of the most important factors in coping with challenges and recovering from adversity (Mira et al., 2023). Sports participation of people with disabilities has shown implication on resilience, especially on access to social support, opportunities and meaningful social experiences (Machida et al., 2013). Social support for athletes with disabilities is extremely relevant to improving their career and well-being. Sport experiences provide an improvement in social skills, which in turn consequently improves well-being and social support (Crawford et al., 2015; Fiorilli et al., 2016; Haslett et al., 2017; Atkinson and Martin, 2020; Aitchison et al., 2021; Mira et al., 2022; Monton et al., 2022). Thus, the aim of our study is to understand the association between social support, resilience and positive affect and negative affect, satisfaction with life, in athletes with disabilities who play federated sport.

### Present study

**Figure 1:**
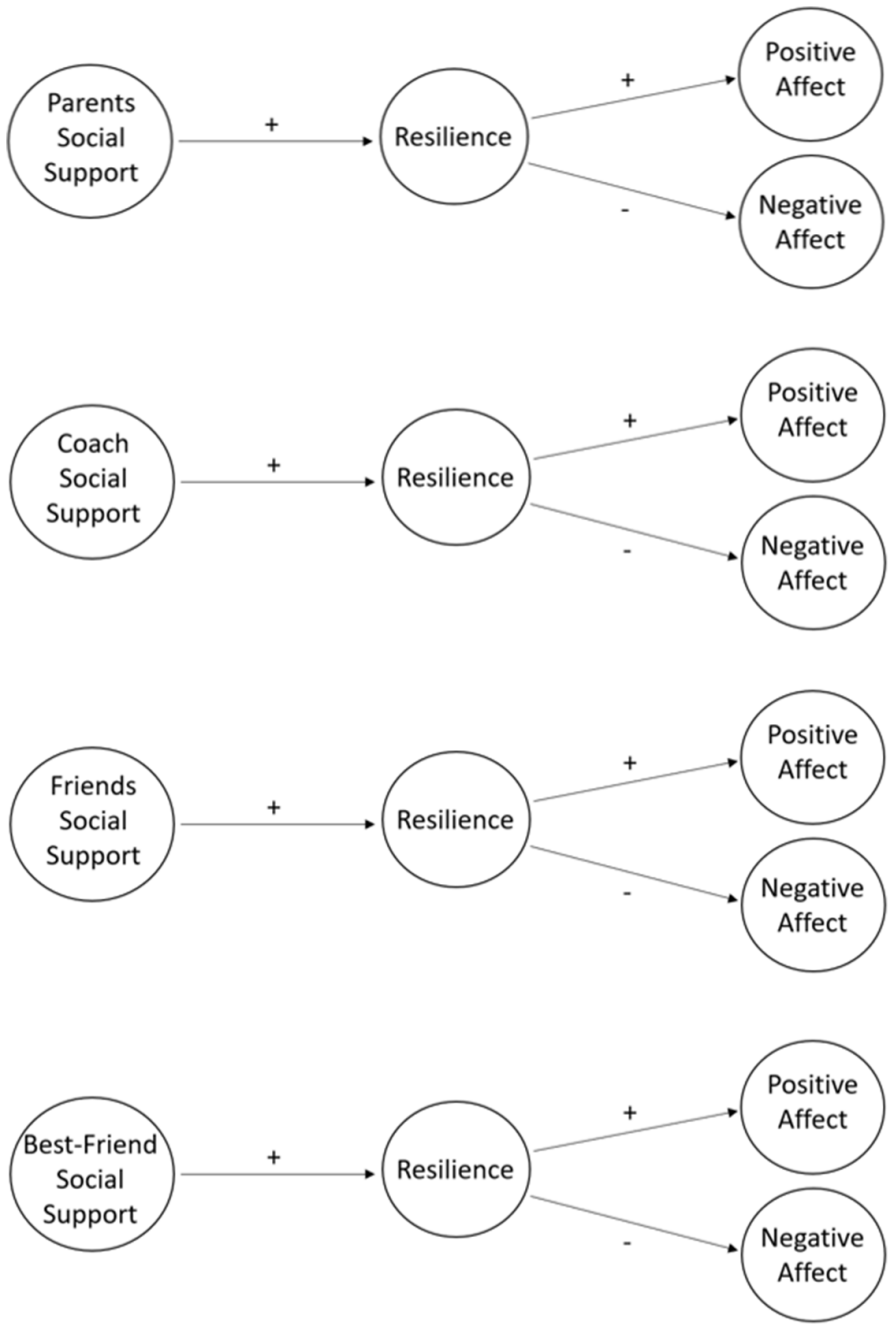
Hypothesized model applied to four types of social support.

Social support from parents, friends, best friend, and coach is fundamental for the sport practice of people with disabilities (Galli and Vealey, 2008; Mira et al., 2022, 2023). These social supports are crucial for the resilience process of these athletes as social support has been pointed out as one of the most important factors to deal with challenges and recover from adversity (Bicalho and Noce, 2019; Mira et al., 2022, 2023). Additionally, the association between resilience and well-being in athletes with disabilities has been demonstrated (Machida et al., 2013; Martin et al., 2015; Sikorska and Gerc, 2018; Atkinson and Martin, 2020; Martin et al., 2022; Mira et al., 2022). Although these variables have already been studied with disabled athletes, this study tries to analyze the relationships of these variables in four models. This study is part of a global project that, in a previous study (Mira et al., 2022), already characterized the Portuguese team present at the Tokyo Paralympic Games, regarding these variables (social support, resilience and affect). However, that study only sought to characterize and analyze associations between the variables, and in a very specific sample of high-performance athletes with disabilities. Thus, the present study intends to analyse the association between types of social support, resilience, and subjective well-being (life satisfaction, positive affect, and negative affect) in a sample of athletes with disabilities who play federated sport (with different competitive levels and sporting experience), according to the model shown in the figure below. This study will allow us to understand the importance of the role that parents, coaches, friends and best friends can have in the practice of sport for people with disabilities (Figure 1).

Based on this assumption, this study aimed to analyze the following hypotheses:

- Parental social support is positively associated with resilience in athletes with disabilities, as suggested by the literature (Machida et al., 2013; Powell and Myers, 2017; Atkinson and Martin, 2020; Mira et al., 2022, 2023);
- Coach social support is positively associated with resilience in athletes with disabilities, as previously reported in the literature (Machida et al., 2013; Powell and Myers, 2017; Atkinson and Martin, 2020; Mira et al., 2022, 2023);
- Social support from friends is positively associated with resilience in athletes with disabilities, as suggested in previous studies (Machida et al., 2013; Powell and Myers, 2017; Atkinson and Martin, 2020; Mira et al., 2022, 2023);
- Best friend social support is positively associated with resilience in athletes with disabilities, in line with previous literature indicators (Martin and Smith, 2002; Martin, 2006; Atkinson and Martin, 2020; Mira et al., 2023);
- Resilience is positively associated with levels of positive affect in athletes with disabilities, as suggested in previous studies with this population (Mira et al., 2022, 2023);
- Resilience is negatively associated with negative affect levels in athletes with disabilities, as suggested by the literature (Mira et al., 2022, 2023).

## Materials and methods

### Study design and procedures

For present study, it was defined, as an inclusion criterion, athletes with disabilities who practice competitive sports in Portugal.

The study protocol explained the objectives of the study, guaranteed the principle of confidentiality, and assumed the acceptance of informed consent to proceed with the application of the questionnaires. Respondents were fully informed about the aim of the study, the procedures for data the voluntary participation. They were also informed that could quit from the study at any time. Participants did not receive compensation for their participation.

The questionnaires were applied in one go via a Google form (between October 2021 and January 2022) and disseminated by athletes with the support of sports federations, clubs, and coaches. Coaches of athletes with visual impairments and with intellectual disabilities were asked to help athletes complete the questionnaires.

After applying the questionnaires, we collect the information and process the data in the computer programs (IBM SPSS STATISTICS v.27.). Each questionnaire evaluated four domains: sociodemographic data, life satisfaction, positive and negative affect, resilience, and social support. The sociodemographic questions were developed specifically for this study, having been reviewed by 4 experts. The other 4 questionnaires are instruments already validated for the Portuguese population.

This study was approved by the ethics committee of the University of Beira Interior (CE-UBI-Pj-2018-076).

### Participants

The study involved 105 athletes with disability from the Portuguese teams aged between 13 and 61 years, mean age of 32 ± 12.35 years, with 78 men (74.3%) with a mean age of 34 ± 13.13 years and 27 women (25.7%) with mean age 30 ± 9.28 years.

Of the 105 athletes, 75 have physical disabilities, 23 have intellectual disabilities, 5 have visual impairments and 2 have multiple disabilities, diagnosed according to the criteria of the International Classification of Functioning (World Health Organization, 2001).

The sample consists of athletes from 13 modalities: 1 of futsal, 1 of goalball, 1 of judo, 1 of ballet, 1 of paradressage, 2 of paracanoeing, 2 of badminton, 3 of table tennis, 10 of athletics, 14 of Boccia, 17 of wheelchair basketball, 18 of wheelchair handball and 34 of paraswimming.

The number of weekly trainings of these athletes varies between one training per week (5.7%), two training per week (20%), three training per week (20%), four training per week (10.5%), five workouts per week (7.6%) and more than five workouts per week (36.2%).

Of these athletes, 29.5% have been practicing the sport for 4 to 7 years, 22.9% for 8 to 11 years and 47.6% have been practicing the sport for 12 years or more. Most athletes (42.9%) train between 2 and 6 h a week, followed by those who train between 11 and 14 h (21.9%), those who train between 7 and 10 h (16.2%), between 15 and 18 h (10.5%), between 19 and 22 h (5.7%) and, finally, those who train more than 22 h (2.9%). Power of sample size has been calculated through Soper (2023) online calculator, following Westland (2010) recommendations. At the same time, based on imput parameters were included: anticipated effect size: 0.4; desired statistical power level: 0.8; number of latent variables: 4; number of observed variables: 20; probability level: 0.05; recommended minimum sample size: 100.

### Measures

#### Sociodemographic characterization

Participants were invited to a sociodemographic questionnaire that focuses on the characterization of issues such as gender, age, time of practice, modality, weekly frequency, weekly training volume and reasons for practicing.

#### Social support

We measured athletes’ with disabilities perceptions of the social support provided by parents, coach, friends and best friend with an adaptation of the Friend Support Scale (α > 0.7) (Jago et al., 2009). Four items were created according to support group as follows: “how often your parents?”; “how often your coach?”; “how often your friends?” and “how often your best friend?”

Participants responded to these four statements: (1) encourage you to exercise or play sports, (2) exercise or play sports with you, (3) tell you that you are doing well in exercise or sports and (4) watch you take part in exercise or sports?.” All items were answered on a four-point scale ranging from 1 (“Strongly disagree”) to 4 (“Strongly agree”).

The scale of support from friends has been used previously in other studies, with acceptable reliability for the same age and language group (Lopes et al., 2015; Monteiro et al., 2021). A confirmatory factor analysis (CFA) of this scale provided an acceptable fit to the data as follows: Coach: (χ^2^ = 16.50; SRMR = 0.031; B-Sp = <0.001; RMSEA = 0.075 [90%CI = 0.059, 0.086]; TLI = 0.946; CFI = 0.976); Parents: (χ^2^ = 52.10; SRMR = 0.062; B-Sp = <0.001; RMSEA = 0.056 [90%CI = 0.049, 0.076]; TLI = 0.916; CFI = 0.926); Friends: (χ^2^ = 7.19; SRMR = 0.034; B-Sp = <0.001; RMSEA = 0.059 [90%CI = 0.038, 0.816]; TLI = 0.978; CFI = 0.989); Best-Friend: (χ^2^ = 10.15; SRMR = 0.057; B-Sp = <0.001; RMSEA = 0.061 [90%CI = 0.047, 0.961]; TLI = 0.939; CFI = 0.953).

#### Subjective well-being

The Positive and Negative Affect Shedule (for positive affect α = 0.85; for negative affect α = 0.91) (PANAS; Watson et al., 1988) in the reduced Portuguese version, by Galinha et al. (2013), consisting of 10 items (five items for positive affect: “inspired,” “alert,” “excited,” “enthusiastic” and “determined” and five items for negative affect: “fear,” “worried,” “nervous,” “scared” and “perturbed”) that are answered on a Likert-type scale, with 5 levels, ranging from 1 (“Not at all or very slightly”) to 5 (“Extremely”).

#### Resilience

Finally, to assess resilience, we used the Brief Resilience Scale (BRS, α = 0.80–0.91) (Smith et al., 2008), in its Portuguese version by da Silva-Sauer et al. (2021). Consisting of 6 items (e.g., “I tend to recover quickly after difficult situations”) that are answered on a Likert-type scale, with 5 levels, ranging from 1 (“I totally disagree”) to 5 (“I totally agree”). A confirmatory factor analysis (CFA) of this scale provided an acceptable fit to the data as follows: (χ2 = 78.99; SRMR = 0.061; B-Sp = <0.001; RMSEA = 0.07 [90%CI = 0.067, 0.112]; TLI = 0.909; CFI = 0.922).

### Data analysis

Means, standard deviation and Pearson’s r bivariate correlations were calculated for all studied variables in IBM SPSS STATISTICS v.27. In terms of Pearson’s bivariate correlation the following cut-off values were used to check the strength of associations: small effect (0.1–0.3); medium effect (0.3–0.5) and large effect (>0.5) (Cohen, 1988). In addition, as suggested by Kline (2016), a two-step approach trough maximum likelihood estimation method was performed in IBM SPSS AMOS (version 27.0). First, the Confirmatory Factor Analysis (CFA) was performed to test the psychometric properties and data adjustment of the measurement model. Therefore, convergent validity was assessed via average variance extracted (AVE), considering values higher than or equal 0.50 as adequate (Fornell and Larcker, 1981). Discriminant validity was estimated through the square correlations between factors, and it was considered adjusted when the square correlations were below the AVE of each factor (Hair et al., 2019). Additionally, the internal consistency of each of the latent variables under study was calculated, from the composite reliability (Raykov, 1997), assuming as a cut-off value for adequacy coefficients, ≥0.70 (Raykov, 1997; Hair et al., 2019). Second, a structural model was established to test the hypothesis. The model’s fit for both the measurement model and the structural model was observed through the traditional goodness-of-fit indexes. Specifically, we used the Comparative Fit Index (CFI) and Tucker-Lewis Index (TLI) and the absolutes of the Standardized Root Mean Residual (SRMR) and Root Mean Square Error of Approximation (RMSEA) with a confidence interval (CI 90%), as recommended by several authors (Marsh et al., 2004; Byrne, 2016; Kline, 2016; Hair et al., 2019) and with the following adopted cut-off values: CFI and TLI ≥ 0.90; RMSEA and SRMR ≤0.08 (Marsh et al., 2004; Byrne, 2016; Kline, 2016; Hair et al., 2019). Standardized direct and indirect effects on the dependent variable were also analyzed. The independent variables are social support provided by the friend, best-friend, parents, and coaches. Dependent variables are positive and negative affect and resilience operate as a possible mediator. The significance of direct and indirect effects was analyzed using a bootstrap resampling procedure (1,000 bootstrap samples), through a 95% CI. The indirect effect was considered significant (≤ 0.05) if the 95% CI did not include zero (Williams and MacKinnon, 2008). We chose to consider confidence intervals rather than the probability of significance (value of p) due to recent evidence of mediation without a significant relationship between variables (Hayes, 2018).

## Results

An inspection of the data revealed that no missing values or outliers, univariate and multivariate were detected. Item-level descriptive statistics indicated no deviations from univariate normality because skewness and kurtosis assumptions of the data distribution were comprised between −2 and +2 and −7 and +7, respectively (Hair et al., 2019). Mardia’s coefficient for multivariate kurtosis exceeded expected values (5.0) for all models under analysis in terms of assumption of multivariate normality (Byrne, 2016). Therefore, the Bollen-Stine bootstrap on 2000 samples was employed for subsequent analysis (Nevitt and Hancock, 2001).

Descriptive statistics showed that the participants presented scores above midpoint for all variables, except negative affect in all models under analysis. Looking at bivariate correlations, positive and negative significant associations were found between resilience and positive and negative affect, respectively. These associations were consistent in all models. It is important to note that, in models of SS-C and SS-F a positive and significant association was observed between social support and positive affect. As seen by the composite reliability (CR) coefficients, each factor showed scores above the cut-off (>0.70), revealing adequate internal consistency. Based on the results of the measurement model and reliability analysis, convergent and discriminant validity were calculated. Convergent validity was achieved, since the AVE scores were above the acceptable cut-off values, as seen in Table 1. According to the squared correlations and AVE scores, all factors demonstrated adequate discriminant validity since the squared correlations of each latent variable were lower than the AVE scores in each latent variable. The results provide preliminary support to conduct Structural Equation Model (SEM) analysis and examine the direct effects of social support provided by best-friends, coach, friends and parents on resilience and positive and negative affect. In addition, indirect effect between social support provided by best-friend, coach, friends and parents and positive and negative affect via resilience can also be analyzed in this way.

**Table 1:** Descriptive statistics, bivariate correlations, average variance extracted, and composite reliability coefficents.

Variables	*M*	SD	1	2	3	4	AVE	CR
*Model SS-BF*								
1. SS-BF	3.17	0.80	1	–	–	–	0.67	0.87
2. Resilience	3.42	1.02	−0.11	1	–	–	0.57	0.76
3. PA	3.78	0.86	0.18	0.28**	1	–	0.62	0.79
4. NA	1.71	0.73	0.08	−0.37**	0.09	1	0.58	0.78
*Model SS-C*								
1. SS-C	3.48	0.55	1	–	–	–	0.69	0.85
2. Resilience	3.42	1.02	0.05	1	–	–	0.58	0.82
3. PA	3.78	0.86	0.22*	0.28**	1	–	0.61	0.82
4. NA	1.71	0.73	−0.03	−0.37**	0.09	1	0.57	0.73
*Model SS-F*								
1. SS-F	3.13	0.76	1	–	–	–	0.69	0.87
2. Resilience	3.42	1.02	−0.03	1	–	–	0.57	0.74
3. PA	3.78	0.86	0.30**	0.28**	1	–	0.62	0.65
4. NA	1.71	0.73	0.18	−0.37**	0.09	1	0.59	0.75
*Model SS-P*								
1. SS-P	2.81	0.84	1	–	–	–	0.66	0.88
2. Resilience	3.42	1.02	−0.19	1	–	–	0.56	0.87
3. PA	3.78	0.86	0.13	0.28**	1	–	0.66	0.72
4. NA	1.71	0.73	0.06	−0.37**	0.09	1	0.68	0.74

M, mean; SD, standard deviation; SS-BF, social support provided by best friend; SS-C, social support provided by coach; SS-F, social support provided by friends; SS-P, social support provided by parents; PA, positive affects; NA, negative affects; AVE, average variance extracted; CR, composite reliability. *p < 0.05; **p < 0.01.

The CFA measurement model including the social support provided by the best friend, coach, friends and parents, resilience and positive and negative affect displayed adequate fit to the data in each sample (see model 1, 2, 3, and 4 in Table 2).

**Table 2:** Goodness-of-fit indexes.

Model	χ^2^	df	χ^2^/df	B-Sp	CFI	TLI	SRMR	RMSEA	CI90%
1. CFA – SS-BF	136.41	105	1.29	0.313	0.947	0.935	0.076	0.061	0.034–0.085
2. CFA – SS-C	116.93	105	1.11	0.566	0.971	0.964	0.062	0.043	0.001–0.070
3. CFA – SS-F	126.05	105	1.20	0.355	0.959	0.950	0.066	0.052	0.018–0.077
4. CFA – SS-P	124.03	105	1.18	0.372	0.962	0.954	0.067	0.051	0.012–0.076
5. SEM – SS-BF	145.13	108	1.34	0.263	0.939	0.927	0.071	0.065	0.039–0.087
6. SEM – SS-C	130.46	108	1.20	0.436	0.954	0.946	0.072	0.053	0.020–0.077
7. SEM – SS-F	142.35	108	1.31	0.224	0.928	0.939	0.080	0.063	0.036–0.086
8. SEM – SS-P	132.83	108	1.22	0.299	0.954	0.945	0.077	0.055	0.024–0.079

CFA, confirmatory factor analysis; SEM, structural equation modeling; χ^2^, chi-square; df, degrees of freedom; gl/df, normalized chi-square; B-Sp, Bollen-Stine level of significance; CFI, comparative fit index; TLI, Tucker Lewis Index; SRMR, standardized root mean square residual; RMSEA, root mean square error of approximation; CI90%, confidence interval at 90% for RMSEA.

The results from the SEM analysis showed that the structural model in each model provided acceptable fit to the data as seen in Table 2 (see model 5, 6, 7, and 8 in Table 2). Positive and significant associations were observed among resilience and positive affect and a negative and significant associations were observed between resilience and negative affect. The associations between social support from best-friends, coach, friends, and parents were not significant. In addition, the indirect effects between social support from best-friends, coach, friends, and parents and positive and negative affect via resilience were not significant, as seen in Table 3.

**Table 3:** Direct and indirect regression paths.

Regression path	Direct				Indirect		
	β	CI95%	*p*		β	CI95%	*p*
*Model SS-BF*				*Model SS-BF*			
SS-BF → RESIL	0.06	−0.272–0.327	0.766	SS-BF → PA	0.01	−0.063–0.152	0.685
RESIL → PA	0.30	0.050–0.565	0.020	SS-BF → NA	−0.02	−0.154–0.096	0.676
RESIL → NA	−0.38	−0.619; − 0.145	0.005	–	–	–	–
*Model SS-C*				*Model SS-C*			
SS-C → RESIL	0.04	−0.236–0.271	0.845	SS-BF → PA	0.01	−0.051–0.120	0.728
RESIL → PA	0.30	0.055–0.569	0.017	SS-BF → NA	−0.01	−0.121–0.089	0.760
RESIL → NA	−0.38	−0.630; −0.147	0.005	–	–	–	–
*Model SS-F*				*Model SS-F*			
SS-F → RESIL	0.14	−0.049–0.340	0.894	SS-BF → PA	−0.04	0.027–0.118	0.063
RESIL → PA	0.29	0.043–0.548	0.020	SS-BF → NA	0.06	0.069–0.327	0.074
RESIL → NA	−0.39	−0.622; −0.142	0.003	–	–	–	–
*Model SS-P*				*Model SS-P*			
SS-P → RESIL	0.01	−0.251–0.308	0.149	SS-BF → PA	0.003	−0.063–0.146	0.847
RESIL → PA	0.30	0.044–0.559	0.024	SS-BF → NA	−0.004	−0.125–0.108	0.867
RESIL → NA	−0.38	−0.621; −0.144	0.004	–	–	–	–

SS-BF, social support provided by best friend; SS-C, social support provided by coach; SS-F, social support provided by friends; SS-P, social support provided by parents; PA, positive affects; NA, negative affects; RESIL, resilience; β, standardized coefficient; CI95%, confidence interval at 95%; p, level of significance.

## Discussion

This study aimed to analyze the associations between types of social support, resilience, and subjective well-being (life satisfaction, positive affect, and negative affect) in a sample of athletes with disabilities. The model was analyzed for the four actors of social support studied, the coach, parents, friends, and best friend.

According to the results, athletes with disabilities presented values above the midpoint for the scales that assess resilience and positive affect and values below the midpoint for the scale that assesses negative affect in the four models of social support analyzed. These results seem to agree with the literature, particularly by Mira et al. (2022), that found that Portuguese Paralympic athletes have high values of life satisfaction, high positive affect, low negative affect, and good levels of resilience.

The results reveal that hypotheses (a), (b), (c), and (d) are not confirmed, since the associations between social support and resilience levels were not significant for any of the sources (parents, friends, best friend, and coach). In addition, the indirect effects between social support from parents, friends, best friend and coach and positive affect and negative affect through resilience were not significant. Contradictory to some studies that have analyzed these variables and argue that to develop mentally strong characteristics and behaviors, athletes in general may benefit from exposure to highly demanding situations in a supportive environment (Powell and Myers, 2017). These include social support from family, coach, peers, and those around them, crucial to their responses in the face of the adversities they encounter (Bicalho and Noce, 2019). Which, in turn, are necessary to know their own limitations and vulnerabilities, finding their own internal strengths and improving levels of well-being through actively combating these adversities (Fredrickson, 1998). Concerning the found associations the results show that, in the models of social support of the coach and friends, a positive and significant association was observed between social support and positive affect. In a previous study conducted in paralympic athletes, positive affect was associated with social support from parents and friends, although the coach presented the value of greatest influence on the athlete, followed by friends, best friends and at last, parents (Mira et al., 2022). These results seem to demonstrate that coach support is more important for federated disabled athletes than specifically for elite (Paralympic) athletes, in contrast to parents. The support of friends has a consensus in its importance for both federated athletes with disabilities and paralympic athletes, which is in line with the literature that considers friendship an important source of social support and influence for the practice of sports (Haye et al., 2011). The origin of social support is extremely important for access to sports practice. However, it does not necessarily have to be positively and significantly associated with resilience or subjective well-being.

The results also show that for the four models analyzed (parents, coach, friends and best friend), there is a direct effect of the types of social support provided with resilience, positive and negative affect. There is also an indirect effect between types of social support and affect (positive and negative) through resilience. Therefore, contrary to what we had considered [hypotheses (a), (b), (c), and (d)], the different types of social support did not show a significant association with the levels of resilience. These results do not seem to be in line with some with the literature, that highlight sociocultural influences as crucial for the resilience process in athletes (Galli and Vealey, 2008). In the study by Li et al. (2021) investigating the main and interactive relationships of social support and resilience on individual mental health during the COVID-19 pandemic across three age groups: emerging adults, adults, and older adults, they identified five social support profiles, and the patterns of potential profiles were similar across all groups. However, the distribution of the categories in the five profiles was significantly different between the age groups. Considering the different age groups presented in our sample, this could be a possible explanation. It would be interesting to explore these data by age group and a much larger sample. On the other hand, it is important to remember the role that types of social support plays in the participation in sport by people with disabilities, as evidenced by different studies (Machida et al., 2013; Crawford et al., 2015; Aitchison et al., 2021; Mira et al., 2022). In the same sense, the origin of this types of social support, it is important the standard adjustment of the athlete’s support throughout his/her career according to his/her changing needs, be it accessibility, disability condition, challenges inherent to the practice of sport (Rees and Hardy, 2000).

Hypotheses (e) and (f) were confirmed, with a positive and significant association between resilience and positive affect and a negative association between resilience and negative affect. The results show that, more than the perceived social support itself, resilience seems to have a preponderant weight in the consequence of sports practice, in this case subjective well-being, in its emotional component (positive and negative affect). This result is particularly relevant if we consider the importance of this emotional dimension of well-being, since the literature has shown, in general, that positive emotions can function as resources for coping with adversity (Fredrickson, 1998; Jaafar et al., 2014). Fredrickson (2001) explains the importance of positive affect in predicting resilience through the broaden-and-build theory. The author argues that an emotion begins with a person own conscious or unconscious appraisal of the significance of an antecedent event for him or her. People with experiences of positive affect are better able to engage and participate in activities in their environment. Affect represents accessible conscious feelings. According to this theory, certain discrete positive emotions, such as joy, interest, satisfaction, pride and love, share the ability to momentarily broaden the thought/action repertoire and build lasting personal resources, evolving from physical and intellectual resources to social and psychological resources. Positive emotions make people feel good in the present, and their effects broaden thinking, increasing the likelihood that people will feel good in the future. They increase people thought/action repertoire, undo persistent negative emotions, stimulate psychological resilience and, by building psychological resilience, trigger upward spirals that increase well-being.

The literature tells us that the study of resilience has been widely carried out with parents and family members of people with disabilities and that the findings have been quite positive although they are not directly related to people with disability. However, the sources of social support are crucial actors in the access to sports practice of these people (Palancı, 2017; Halstead et al., 2018; Mohan and Kulkarni, 2018; Rajan et al., 2018). Therefore, it would be interesting to analyze the levels of resilience of parents, friends, best friend, and coach in the models themselves.

These results of our study agree with the study previously conducted in a sample of paralympic athletes (Mira et al., 2022), where it was noted that the negative association between resilience and negative affect seems to indicate a possible blocking effect of resilience to emotionally negative experiences of athletes (Ryff and Singer, 2003; Hammond, 2014; Hariharan et al., 2014; Mira et al., 2022). Other studies have proven the association between resilience and subjective well-being in athletes with disabilities (Martin et al., 2015; Sikorska and Gerc, 2018; Atkinson and Martin, 2020; Silva et al., 2020; Mira et al., 2023), which is in accordance with the importance of this variable in this population that, in a given risk situation, one can react in a vulnerable way, with a negative affect response, or in a resilient way, with a positive affect response (Fredrickson, 1998).

The analysis of all models of social support showed a direct effect on resilience and positive and negative affect. Literature tells us that exposure of disabled athletes to highly demanding and socially supported situations benefits them in developing resilient characteristics and behaviors (Machida et al., 2013; Mira et al., 2023).

The results of the present study may constitute an important contribution to practice, particularly for all those working in the context of adapted sport, as they highlight the importance of monitoring these variables throughout the process. It becomes fundamental that types of social support acts as a teamwork that supports in the various challenges and tasks inherent and adjusted to the characteristics and needs of athletes with disabilities (Crawford et al., 2015) and, therefore, the sources of types of social support should be multiple, from family, therapists, colleagues, coaches, among others (Machida et al., 2013). Types of social support provided by a multidisciplinary team presents an essential role in the development and improvement of athletes’ training and performance. The social support of family, friends and other performance agents are considered the necessary and indispensable support for the provision of mental health care and happiness in general (Sheridan et al., 2014). Resilience seems to play an extremely relevant role and to have an impact on the well-being perceived by athletes, and should be the subject of attention and should be a variable to be enhanced in the context of sport. Sport as an environment that exposes athletes to the risk, needs and stress inherent in the competitive environment, allows athletes with disabilities to strengthen their personal and social resources, as well as their positive characteristics and social support network, which will allow them to overcome adversity successfully, with above-average levels of resilience.

Despite the results of this study, there are some limitations that should be taken into account in future studies. Although our sample fulfils the criteria, it is relatively small, and future studies should consider recruiting larger samples. On the other hand, other variables that could play an important role in this process were not analyzed, such as the type of disability or sport played and the effect of age. A longitudinal analysis would also be important. At the same time, it will also be important to try in order to try to validate the Brief Resilience Scale for this population in the future.

## Conclusion

The present findings seem to indicate that the effect of social support provided by the best friend, coach, friends, and parents had a direct effect on resilience and positive and negative affect. We also found a positive and significant association between resilience and positive affect and a negative association between resilience and negative affect. The strongest relationship in the variables studied was found between resilience and affect, with no relationship being verified between the sources of social support and resilience or affect, as hypothesized. For this group of athletes with disability, more than the social support they may have or may feel, resilience proved to be very important for the consequence of sports practice in terms of subjective well-being.

### Transparency statement

This study is part of a global research project on Portuguese athletes with disabilities. Thus, in a first study we sought to characterize the population of high-performance athletes, namely the Portuguese team that was present at the Tokyo 2020 Paralympic Games (Mira et al., 2022), that aimed characterize the Portuguese delegation at the Tokyo 2020 Paralympic Games through sociodemographic and psychosocial variables (positive and negative affect, life satisfaction, resilience, and social support). However, with the present work we aimed to reach a larger sample with different characteristics. Thus, keeping the paralympic athletes already studied, we also added athletes with different years of practice and with different competitive levels. Moreover, in this work, we did not seek only a descriptive analysis but an analysis in a single model that could explain the associations between the different variables.

## Data availability statement

All relevant data is contained within the article: The original contributions presented in the study are included in the article, further inquiries can be directed to the corresponding author.

## Ethics statement

The studies involving humans were approved by the Ethics committee of the University of Beira Interior (CE-UBI-Pj-2018-076). The studies were conducted in accordance with the local legislation and institutional requirements. The participants provided their written informed consent to participate in this study.

## Author contributions

TM: Conceptualization, Data curation, Investigation, Methodology, Writing – original draft. MJ: Data curation, Formal analysis, Methodology, Software, Visualization, Writing – review & editing. AC: Conceptualization, Data curation, Funding acquisition, Methodology, Project administration, Validation, Writing – review & editing. DM: Conceptualization, Data curation, Formal analysis, Investigation, Methodology, Project administration, Software, Writing – review & editing. SD: Data curation, Investigation, Visualization, Writing – review & editing. RM: Resources, Validation, Writing – review & editing. RA: Conceptualization, Formal analysis, Investigation, Methodology, Project administration, Supervision, Validation, Visualization, Writing – original draft, Writing – review & editing.
